# *Griflola frondosa* (GF) produces significant antidepressant effects involving AMPA receptor activation in mice

**DOI:** 10.1080/13880209.2016.1235590

**Published:** 2016-12-10

**Authors:** Hongkun Bao, Pengzhan Ran, Lijuan Sun, Weihong Hu, Hongliang Li, Chunjie Xiao, Keming Zhu, Jing Du

**Affiliations:** a School of Medicine, Yunnan University, Kunming, Yunnan, China;; b School of Life Sciences, Yunnan University, Kunming, Yunnan, China

**Keywords:** Medical mushroom, animal behaviour, tail suspension test, forced swim test, polysaccharide

## Abstract

**Context:***Griflola frondosa* (Fr) S.F. Gray (Meripilaceae) (GF) is a medical mushroom, and its regulation of the immune system is of interest for the treatment of mood disorders. α-Amino-3-hydroxy-5-methyl-4-isoxazole-propionic acid (AMPA) receptors are the central mediator for the treatment of depression.

**Objective:** This study examines the antidepressant effects of GF and the role of AMPA in these antidepressant effects.

**Materials and methods:** The CD-1 mice were fed with GF- or *Pleurotus ostreatus* [(Jacq.: Fr) Kumm (Pleurotaceae)] (PO)-containing food for 1 day or 5 days. The antidepressant effects was determined in the tail suspension test (TST), forced swim test (FST), and open field test (OFT). The involvement of AMPA receptors was determined by the application of the AMPA-specific blocker GYKI 52466.

**Results:** Treatments with 20%, 33% or 50% of GF-containing food significantly decreased the immobility time (63.6, 56.9, and 52.0% in TST; and 50.8, 43.2, and 38.2% in FST) after 1 day and (62.3, 51.8, and 52.8% in TST; and 49.5, 45.1, and 40.3% in FST) after 5 days. GF-containing food did not cause hyperactive effects in the OFT. The antidepressant effects of the 33% of GF-containing food (down-to 51.3% in 1-day TST and 46.8% in 5-day FST) were significantly stronger than that of the 33% of PO*-*containing food (down-to 85.5% in 1-day TST and 82.0% in 5-day FST). AMPA-specific blocker GYKI 52466 was able to block the antidepressant effects of the GF-containing food.

**Conclusion:** GF demonstrated the potential as a safe medical food supplement for the patient with depression.

## Introduction

Major depressive disorder (MDD), a common mental disorder, is the leading cause of disability and a major contributor to disease burden in the global population (Ghasemi et al. 2014). Medicinal mushrooms have an established history of use in traditional oriental therapies for the prevention and treatment of diseases (Cui & Chisti 2003). *Griflola frondosa* (Fr) S.F. Gray (GF) (Meripilaceae) is a medical fungus, commonly known as Maitake or Hui-shu-hua. The properties of GF medicinal potentials include various physiological benefits, ranging from enhanced immune systems and decreased blood glucose to improved spleen, stomach and nerve functioning (Shen et al. 2015). Recent evidence has shown that depression is related to immune regulation (Kong et al. 2015; Liu et al. 2015). However, whether GF shows antidepressant effects remains unknown.

Glutamate is thought to mediate ∼80% of the excitatory synaptic transmission in the central nervous system (CNS) (Douglas & Martin 2007; Sanacora et al. 2012). A growing body of evidence suggests that the glutamatergic system might be crucial for the pathophysiology and treatment of depression (Krystal et al. 2002; Musazzi et al. 2013; Pilc et al. 2013; Sanacora et al. 2012). Clinical evidence suggests that the expression of AMPA receptors is decreased in the brain in patients with depression (Beneyto et al. 2007; Duric et al. 2013). In fact, AMPA receptors may be a common downstream pathway for known antidepressants, which act initially on entirely different molecular targets (Bleakman et al. 2007). The use of antagonists that block AMPA receptors has been shown to prevent the antidepressant effects of the fast-acting antidepressant ketamine (Maeng et al. 2008; Autry et al. 2011; Koike et al. 2011), indicating that AMPA receptors may be critical for antidepressant effects.

Based on these premises, we designed a series of behavioural experiments to investigate the antidepressant effects of GF in the animal models of depression and to explore the roles of AMPA receptors in these antidepressant effects. We studied the effects of various GF supplementations in various ratios in animal models using the tail suspension test (TST), forced swim test (FST) and open field test (OFT). To determine whether the antidepressant effects of GF are unique among commonly used medical mushrooms, we selected *Pleurotus ostreatus* [(Jacq.: Fr) Kumm (Pleurotaceae)] (PO) as another mushroom control. In addition, we investigated the possible involvement of AMPA receptors in the antidepressant effects using AMPA-specific antagonist GYKI 52466.

## Materials and methods

### Animals

All animal procedures were carried out in accordance with the Guide for the Care and Use of Laboratory Animals (ISBN: 0-309-05377-3) and were approved by the Institutional Animal Care and Use Committee at Yunnan University, School of Medicine (IACUC: MS201402). Male CD-1 mice (6 weeks; starting weight, 22–26 g; Vital River, Beijing, China) were group housed (*N* = 4/cage) in an animal room with a constant temperature (22 ± 1 °C) and maintained on a 12 h light/dark cycle with constant humidity (55 ± 10%) and free access to water and food.

### Animal behavioural studies

**Figure 1.**
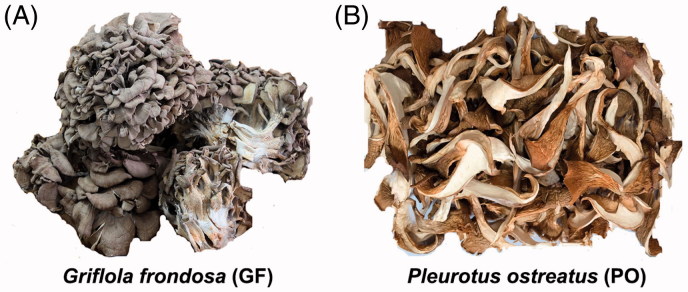
*Griflola frondosa* (GF) and *Pleurotus ostreatus* (PO). (A) *Griflola frondosa* (GF). (B) *Pleurotus ostreatus* (PO).

**Figure 2.**
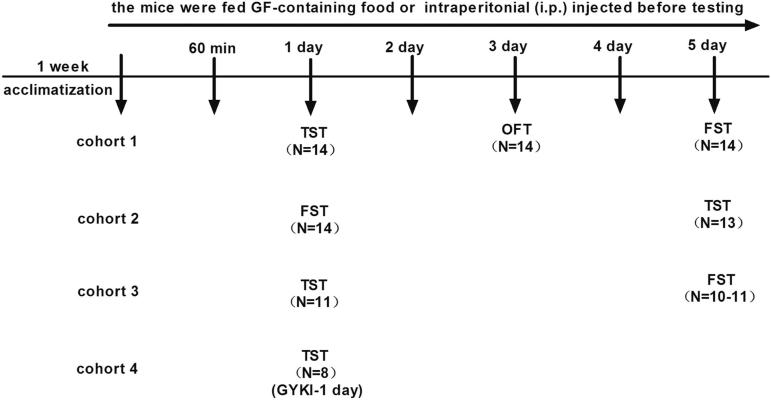
Experimental schedule. Mice were acclimatized for at least 1 week. Four independent cohorts of animals were used to test the antidepressant effects of *Griflola frondosa* (GF). After the animals received the GF-containing food for 24 h, the 1st animal behavioural tests were performed 60 min after drugs or vehicle were administered. The 1st independent cohort of animals underwent the tail suspension test (TST) on the 1st day, open field test (OFT) on the 3rd day and forced swim test (FST) on the 5th day. To confirm the antidepressant effects of GF, the 2nd independent cohort of animals was subjected to the FST on the 1st day and to the TST on the 5th day under similar conditions. The 3rd independent cohort of animals was used to test the antidepressant effects of GF are unique among commonly used medical mushrooms after 1 day treatment in the TST and FST on the 5th day. The 4th independent cohort of animals was used to test whether the α-amino-3-hydroxy-5-methyl-4-isoxazole-propionic acid (AMPA) receptor-specific antagonist GYKI 52466 (GYKI) was involved in the antidepressant effects of GF in the TST.

To examine the antidepressant effects of GF (from Yunnan Wild Fungus Mushroom Flora Co., Ltd., Kunming, China) (Figure 1(A)), CD-1 mice were fed with regular chow or regular chow containing GF. GF-containing food was made from mixing the *Griflola frondosa* powder and regular chow powder in the designated ratio, followed by baking them in the same procedure as making the regular chow. The mice were randomly assigned to three GF experimental groups: low dose of GF-containing food (made from 1 kg of *Griflola frondosa* powder in 4 kg of mouse chow, 1:4 ratio), medium dose of GF-containing food (made from 1 kg of *Griflola frondosa* food powder in 2 kg of mouse chow, 1:2 ratio) and high dose of GF-containing food (made from 1 kg of *Griflola frondosa* powder in 1 kg of mouse chow, 1:1 ratio). The positive control group mice were intraperitonially (i.p.) injected with imipramine (15 mg/kg, in saline; Sigma, St. Louis, MO); mice in the negative control group were i.p. injected with saline (0.9% sterile sodium chloride solution). After the animals received the GF-containing food for 24 h, the drugs or vehicle were i.p. administered. The 1st animal behavioural tests were performed 60 min after drugs or vehicle were administered. The TST was performed on the 1st day, the OFT on the 3rd day, and the FST on the 5th day (Figure 2). To confirm the antidepressant effects of GF, another batch of mice were subjected to the FST on the 1st day and to the TST on the 5th day, respectively, under similar treatment (Figure 2).

To examine whether the antidepressant effects of GF were unique, another group of mice were randomly assigned to four experimental groups: medium dose of GF-containing food (made from 1 kg of *Griflola frondosa* powder and 2 kg of mouse chow, 1:2 ratio), medium dose of *Pleurotus ostreatus* (PO)-containing food (Yunnan Wild Fungus Mushroom Flora Co., Ltd. Kunming, China) (made from 1 kg of *Pleurotus ostreatus* powder and 2 kg of mouse chow, 1:2 ratio) (Figure 1(B)). Positive control mice were i.p. injected with imipramine (15 mg/kg, in saline; Sigma, St. Louis, MO) for the positive control group and with saline (0.9% sterile sodium chloride solution) for mice in the negative control group. Animal behavioural tests were performed 60 min after drugs or vehicle were administered, the TST was performed on the 1st day and the FST on the 5th day (Figure 2).

To examine whether the antidepressant effects of GF could be blocked by GYKI 52466 (a selective noncompetitive AMPA receptor antagonist, TOCRIS Bioscience, R&D, Minneapolis, MN), mice were fed medium dose of GF-containing food (made from 1 kg of *Griflola frondosa* powder and 2 kg of mouse chow, 1:2 ratio) followed by GYKI 52466 (15 mg/kg in 16% DMSO/84% saline) injection. GYKI 52466 or vehicle were administered 30 min prior to the TST (Figure 2) (Gould et al. 2008; Farley et al. 2010). For each drug treatment, the control mice received the respective vehicle alone.

### Tail suspension test (TST)

The TST was performed according to a previously described procedure (Cryan et al. 2005a) with minor modifications. Mice were suspended by the tail 50 cm above the floor by adhesive tape placed ∼2 mm from the tip of the tail. During the test, no mice climbed their tails. Each mouse was individually videotaped during a 6 min test session. The immobility time was quantified by a naive observer for the last 4 min of the 6 min session.

### Open field test (OFT)

An activity chamber (60 × 60 × 30 cm) with a black floor was divided into 16 squares of equal area (15 × 15 cm) by white lines and used to study GF-induced locomotor hyperactivity. Three days after treatment with regular chow or regular chow containing GF, mice were placed in the centre of the chamber and their behaviours were recorded for 60 min. The total distance travelled and the distance travelled in the centre area (the 4-square area in the middle of the chamber) were analysed by the ANY-maze system (Stoelting, Wood Dale, IL).

### Forced swim test (FST)

The FST was carried out according to the previously described procedures (Borsini & Meli 1988) with minor modifications. Mice were placed in a cylinder (Φ = 20 cm) with water (temperature between 23 ± 1 °C) 20 cm in depth. Mice were videotaped during a 6 min test session and were later analysed for mobility for the final 4 min. Mobility was defined as any movement beyond what was necessary to maintain their head above water. Immobility time was quantified by a naive observer.

### Statistical analysis

All data were analysed by one-way analysis of variance (ANOVA) and *post hoc* Tukey’s tests and presented as the mean ± SE via SPSS version 17 (Chicago, IL). Any experimental data value greater than mean plus 2 × standard deviations (SDs) from a group was considered an outlier and was not considered in the analysis. A *p*-value less than 0.05 was considered significant. Figures were generated by GraphPad Prism version 5 software (La Jolla, CA).

## Results

### GF demonstrated significant antidepressant effects in the TST

**Figure 3.**
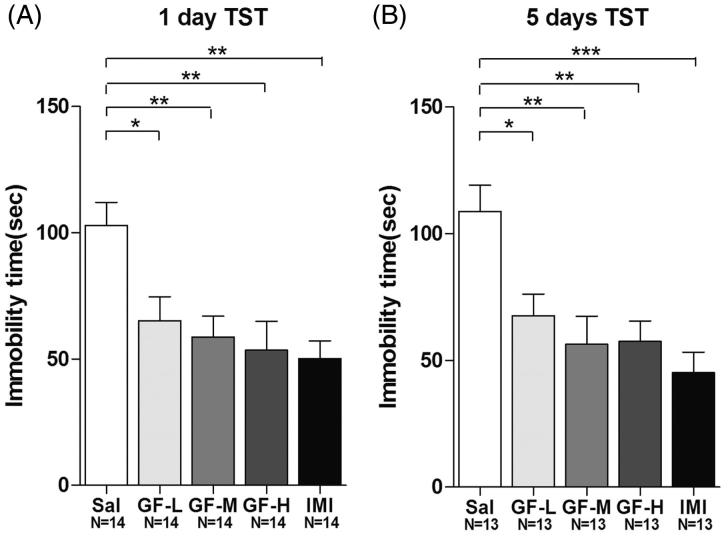
*Griflola frondosa* (GF) demonstrated significant antidepressant effects in the tail suspension test (TST). CD-1 mice were fed with regular mouse chow, a low dose of *Griflola frondosa* (GF powder:mouse chow =1:4, GF-L); a medium dose of *Griflola frondosa* (GF powder:mouse chow =1:2, GF-M); or a high dose of *Griflola frondosa* (GF powder:mouse chow =1:1, GF-H). For the positive control group, mice were i.p. injected with imipramine (15 mg/kg/day, IMI). Mice in the negative control group were i.p. injected with saline (Sal). One day or five days after the GF-treated food intake, mice were subjected to the TST. The number of mice per group is indicated in each individual graph. Data were analysed by one-way ANOVA and presented as the mean ± SE (*post hoc* Tukey’s test, *p < 0.05, **p < 0.01, ***p < 0.001). (A) One day after the administration, GF treatment significantly reduced the immobility time in the TST. (B) Five days after the GF administration, GF again significantly reduced the immobility time in the TST.

To investigate whether GF plays a role in regulating depression-like behaviours, 7-week-old CD-1 mice were fed with their regular chow or with a low (1:4), a medium (1:2) or a high (1:1) dose of GF, or i.p. injected with drugs or vehicle. After 1 day of treatment with GF, the mice were subjected to the TST. The data showed that the immobility time in the GF-treated groups were significantly lower than the negative controls (103.0 ± 9.1 s) in a dose-dependent manner, being 65.2 ± 9.5 s (for 1:4), 58.7 ± 8.3 s (for 1:2), and 53.6 ± 11.5 s (for 1:1) in the TST (ANOVA, F(4,65) = 5.418, *p* < 0.01) (Figure 3(A)). The positive control imipramine also demonstrated expected antidepressant effects (50.2 ± 7.1 sec) (Figure 3(A)). To investigate whether GF treatment resulted in longer-term regulation of depression-like behaviours, we fed treated mouse chow (at low, 1:4; medium, 1:2; or high, 1:1 doses) for 5 consecutive days or continued i.p. injections. After 5 days of treatment with GF, mice were subjected to the TST. The data showed that the immobility time in the GF-treated groups were significantly lower than the negative control group (108.8 ± 10.4 s) at 67.8 ± 8.5 s (for 1:4), 56.4 ± 11.1 s (for 1:2), and 57.5 ± 8.1 s (for 1:1) in the TST (ANOVA, *F*(4,60) = 7.009, *p* < 0.01) (Figure 3(B)). Again, the positive control imipramine (45.2 ± 8.1 s) also showed an antidepressant effect (Figure 3(B)).

### GF exerted significant antidepressant effects in the FST

**Figure 4.**
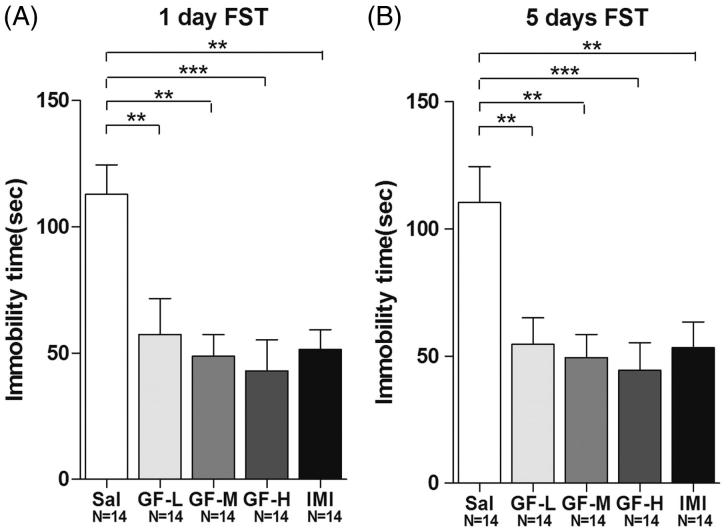
*Griflola frondosa* (GF) treatment demonstrated significant antidepressant effects in the forced swim test (FST). CD-1 mice were fed with their regular mouse chose, a low dose of *Griflola frondosa* (GF powder:mouse chow =1:4, GF-L); a medium dose of *Griflola frondosa* (GF powder:mouse chow =1:2, GF-M); or a high dose of *Griflola frondosa* (GF powder:chow food =1:1, GF-H). For the positive control group, mice were i.p. injected with imipramine (15 mg/kg/day, IMI). Mice in the negative control group were i.p. injected with saline (Sal). One day or five days after the GF-containing food intake, mice were subjected to the FST. The number of mice per group is indicated in each individual graph. Data were analysed by one-way ANOVA and presented as the mean ± SE (*post hoc* Tukey’s test, *p < 0.05, **p < 0.01, ***p < 0.001). (A) One day after the administration, GF treatment significantly reduced immobility time in the FST. (B) Five days after the GF administration, GF also significantly reduced immobility time in the FST.

To confirm the data from the TST further, the FST was performed under similar conditions. After 1 day of treatment with GF, all doses also demonstrated strong antidepressant effects at 57.4 ± 14.2 s (for 1:4), 48.8 ± 8.5 s (for 1:2), and 43.1 ± 12.3 s (for 1:1), compared with the negative control (112.9 ± 11.7 s) group (ANOVA, F(4,65) = 6.518, *p* < 0.01) (Figure 4(A)), in a dose-dependent manner. Similarly, the imipramine group (51.4 ± 7.9 s) also demonstrated antidepressant effects (Figure 4(A)). After 5 days of treatment with GF, all doses also exerted strong antidepressant effects at 54.7 ± 10.4 s (for 1:4), 49.5 ± 9.1 s (for 1:2), and 44.5 ± 10.8 s (for 1:1); compared with the negative control (110.4 ± 14.1 s) (ANOVA, *F*(4,65) = 6.025, *p* < 0.01) (Figure 4(B)) in the FST. The imipramine group (53.4 ± 10.2 s) also showed an antidepressant effect (Figure 4(B)) in the FST after five days of treatment.

### GF did not result in locomotor hyperactivity in the OFT

**Figure 5.**
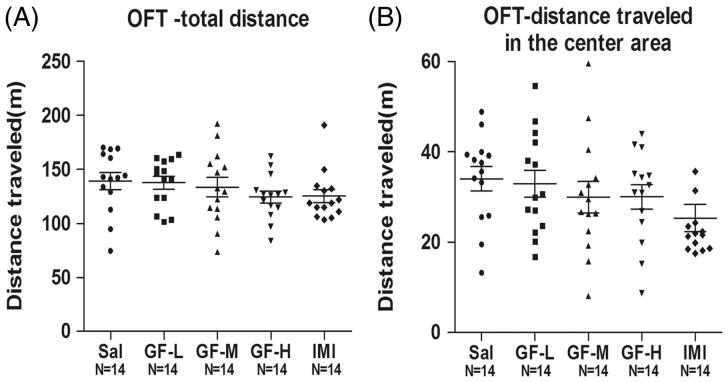
Open field test (OFT) after 3 days of treatment with *Griflola frondosa* (GF). CD-1 mice were fed with their regular mouse chow, a low dose of *Griflola frondosa* (GF powder:mouse chow =1:4, GF-L); a medium dose of *Griflola frondosa* (GF powder:mouse chow =1:2, GF-M); or a high dose of *Griflola frondosa* (GF powder:mouse chow = 1:1, GF-H). For the positive control group, mice were i.p. injected with imipramine (15 mg/kg/day, IMI). Mice in the negative control group were i.p injected with saline (Sal). After 3 days of treatment with GF-containing food, mice were subjected to the OFT. Total distance travelled and the distance travelled in the centre area were determined by an automated tracking system. The number of mice per group is indicated in each individual graph. Data were analysed by one-way ANOVA and presented as the mean ± SE. (A) The total distance travelled in the field after GF treatment. (B) The distance travelled in the centre area of field after GF treatment.

To determine further whether GF causes locomotor hyperactivity, one of the core features of mania-like symptoms, we performed the OFT after 3 days of treatment with the low, medium or high doses GF groups. The total distance travelled showed no significant differences in GF-treated groups compared with the negative control, suggesting that GF did not cause locomotor hyperactivity in mice (ANOVA, *F*(4,65) = 0.9706, *p* = 0.4297) (Figure 5(A)). In support of these data, the distance travelled in the centre area also showed no significant differences in GF-treated groups compared with the negative control group (ANOVA, *F*(4,65) = 1.276, *p* = 0.2884) (Figure 5(B)).

### Increased food intake and normal body weight of mice treated with GF

**Figure 6.**
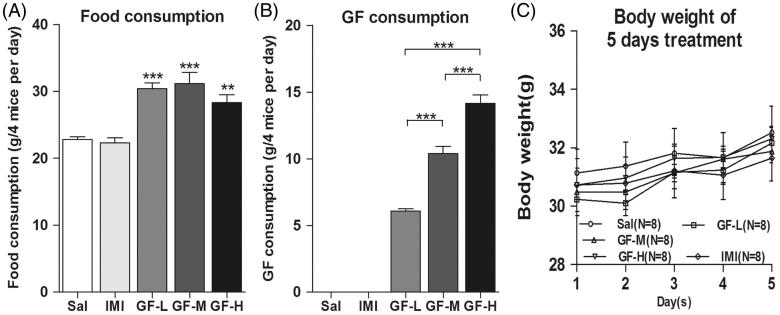
Increased food intake and normal body weight after GF-containing food administration for five days. CD-1 mice were fed with their regular mouse chow, a low dose of *Griflola frondosa* (GF powder:mouse chow =1:4, GF-L); a medium dose of *Griflola frondosa* (GF powder:chow food =1:2, GF-M); or a high dose of *Griflola frondosa* (GF powder:mouse chow =1:1, GF-H). For the positive control group, mice were i.p. injected with imipramine (15 mg/kg/day, IMI). Mice in the negative control group were i.p injected with saline (Sal). After 5 days of drug administration, the amount of food intake and the body weight were measured. Data were analyzed by one-way ANOVA and presented as the mean ± SE (*post hoc* Tukey’s test, *p < 0.05, **p < 0.01, ***p < 0.001). (A) The food intake of mice for the GF-containing food. (B) The actual amount of GF intake of mice for the different dosages. (C) Body weights of the mice after 5 days of GF-containing food treatment.

CD-1 mice were fed with regular mouse food, and various ratios of GF-containing food. We recorded mouse food consumption every day. After 5 days of treatment, the intake data showed that all ratios of GF-food supplementation were higher compared with negative controls (ANOVA, F(4,70) = 15.41, *p* < 0.01) (Figure 6(A)). While it looks like the overall amount of food consumed for the different dosages is quite comparable, there yet may be a mild difference in the consumption. The actual amount of GF consumption showed a significant difference for the different dosages (ANOVA, *F*(2,42) = 69.12, *p* < 0.01) (Figure 6(B)). However, the weights of the mice after 5 days of treatment did not show a significant change (ANOVA, *F*(4,35) = 1.185, *p* = 0.3476) (Figure 6(C)).

### The antidepressant effects of GF are stronger than thePO-treated group

**Figure 7.**
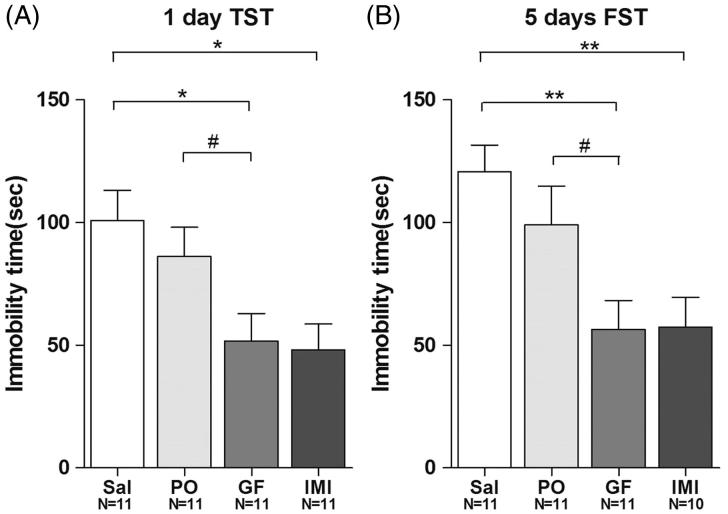
The antidepressant effects of *Griflola frondosa* (GF) are stronger than that of *Pleurotus ostreatus* (PO). CD-1 mice were fed with their regular mouse chow, a medium dose of GF-containing food (GF powder:mouse chow = 1:2), or a medium dose of *Pleurotus ostreatus* (PO) (PO powder:mouse chow = 1:2). For the positive control group, mice were i.p. injected with imipramine (15 mg/kg/day, IMI). Mice in the negative control group were i.p. injected with saline (Sal). After one day of drug administration, mice were subjected to the TST; and after 5 days of drug administration, mice were subjected to the FST. The number of mice per group is indicated in each individual graph. Data were analysed by one-way ANOVA and presented as the mean ± SE (*post hoc* Tukey’s test, *p < 0.05, **p < 0.01, ***p < 0.001; two-tail t-test, #*p* < 0.05, ##*p* < 0.01). (A) After one day of treatment, GF significantly reduced immobility time in the TST but PO did not. (B) After 5 days of treatment, GF significantly reduced immobility time in the FST, but PO did not.

To investigate whether GF plays an important role in regulating depression-like behaviours, 7-week-old CD-1 mice were fed with a medium (1:2) dose of GF and a medium (1:2) dose of PO for 1 day or 5 days. After 1 day of treatment with GF or PO, the mice were subjected to the TST. The data showed that the immobility time in the GF-treated groups (51.7 ± 11.2 s) were significantly lower than the control (100.8 ± 12.3 s) and the PO-treated group (86.2 ± 11.9 s) in the TST (ANOVA, *F*(3,40) = 5.060, *p* < 0.01) (Figure 7(A)). Again, the positive control imipramine (48.1 ± 10.6 s) also showed an antidepressant effect (Figure 7(A)). After 5 days of treatment with GF or PO, the mice were subjected to the FST. The GF-treated group (56.5 ± 11.7 s) demonstrated strong antidepressant effect compared with the negative control (120.8 ± 10.8 sec) and PO-treated group (99.1 ± 15.8 s) (ANOVA, F(3,39) = 6.201, *p* < 0.01) (Figure 7(B)). The imipramine group (57.3 ± 12.3 s) also showed an antidepressant effect (Figure 7(B)). The PO-treated group showed a trend towards an antidepressant effect that did not reach the significance.

### The antidepressant effect of GF was blocked by theAMPA-receptor-specific antagonist GYKI 52466

**Figure 8.**
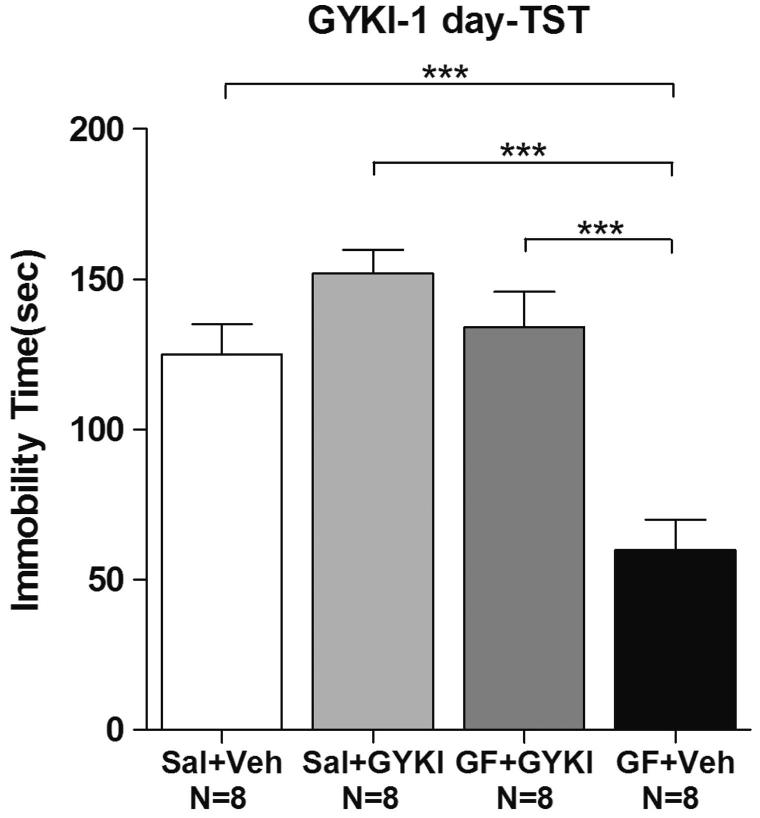
The α-amino-3-hydroxy-5-methyl-4-isoxazole-propionic acid (AMPA) receptor-specific antagonist GYKI 52466 (GYKI) significantly blocked the *Griflola frondosa* (GF)-induced antidepressant effects in the tail suspension test (TST). CD-1 mice were fed mouse chow with a medium dose of GF (GF powder:mouse chow =1:2) or saline (Sal) for 1 day. On the second day, the mice were treated again with GF, and 60 min after GF treatment, GYKI 52466 (15 mg/kg) or vehicle (Veh) was administered 30 min prior to behavioural testing. Then, the CD-1 mice were subjected to the TST. Immobility time were determined. The number of mice per group is indicated in each individual graph. Data were analyzed by one-way ANOVA and presented as the mean ± SE (*post hoc* Tukey’s test, *p < 0.05, **p < 0.01, ***p < 0.001).

We hypothesized that the enhancement of AMPA receptor signalling is critical for the antidepressant effect of GF. AMPA-receptor-specific antagonist GYKI 52466 was used to further investigate this possibility. CD-1 mice were fed with a medium (1:2) dose of GF-containing food for 1 day. GYKI 52466 (15 mg/kg) was administered to the animals. Thirty minutes after GYKI 52466 injection, the mice were subjected to the TST. Treatment with GYKI 52466 almost completely blocked the decrease in immobility time previously observed with GF (Control: 125.0 ± 10.1 s; GYKI: 152.0 ± 7.8 s, GF + GYKI: 134.1 ± 11.7 s, GF: 59.9 ± 10.1 s) (ANOVA, *F*(3,28) = 16.17, *p* < 0.01) (Figure 8), suggesting that enhanced AMPA receptor excitability at the synapses might play an important role in the antidepressant effect of GF.

## Discussion

In this paper, we investigated the antidepressant effects of the medical mushroom GF. We found that (1) GF demonstrated antidepressant effects in the TST and FST after 1-day or 5-day treatments; (2) GF led to no hyperactive effects in the OFT; (3) The antidepressant effects of GF were stronger compared to the other medical mushroom PO; (4) AMPA receptor-specific antagonist GYKI 52466 was able to block the antidepressant effects of GF, suggesting that AMPA receptors are involved in the antidepressant effects of GF.

The antidepressant mechanisms of GF might be related to the immunomodulatory functions of GF. Specifically, it has been proposed that the bioactive polysaccharides or peptides may be responsible for the antidepressant effects of GF. Mushrooms are a popular and valuable food (culinary and medicinal mushroom), low in calories and high in essential amino acids, minerals, vitamins and fibers (Mattila et al. 2002). GF produce substances with potential medical effects, for example, polysaccharides (in particular β-d-glucans) in their cell wall, and GF demonstrated immune regulatory functions (Deng et al. 2009). The major immune-modulating effects of these active substances derived from GF include mitogenicity and the activation of immune cells, such as hematopoietic stem cells, lymphocytes, macrophages, dendritic cells and natural killer cells, resulting in the production of cytokines. Macrophages stimulated by GF products release several inflammatory cytokines, IL-1, IL-6, IL-8, TNF-α, and NO, all of which directly induce tumoricidal activity in macrophages (Adachi et al. 1994; Okazaki et al. 1995; Ishibashi et al. 2001; Sanzen et al. 2001). In some case, macrophages produce their anti-inflammatory effects via the down regulation of iNOS, COX-2, IL-1β, and TNF-α gene expression via the suppression of NF-κB activation (Bai et al. 2005; Kim et al. 2008; Mengoni et al. 2011). Recent evidence has shown that immune cells and their signalling play a major role in the pathophysiology of major depressive disorder (Kong et al. 2015; Liu et al. 2015). There is evidence that the release of neuroactive cytokines, particularly interleukins, such as IL-1β, IL-6 and TNF-α, are altered in mood disorders (Bhattacharya et al. 2016). Moreover, the regulation of microglia and astroglia in central neuroinflammation and their interactions with the peripheral immune system may be involved in the treatment of mood disorders. The TST and FST have become the most widely used models for assessing antidepressant-like activity for antidepressant drug screening. The TST was originally proposed by Steru et al. (1985) as a primary screening test of anti-depressant drugs in mice. The test is based on the fact that animals subjected to the short-term, inescapable stress of being suspended by their tail, will develop an immobile posture. Various antidepressant medications reverse the immobility and promote the occurrence of escape-related behaviour. The TST is a useful test for assessing the behavioural effects of antidepressant compounds and other pharmacological and genetic manipulations relevant to depression (Cryan et al. 2005a). The FST was developed by Porsolt et al. (1977) in the rat and mouse (Porsolt 2000). This test is the most widely used tool for assessing antidepressant activity preclinically. The widespread use of this model is largely a result of its ease of use, reliability across laboratories and ability to detect a broad spectrum of antidepressant agents (Borsini & Meli 1988). It has been shown that the sensitivity of the rat FST to an impressively broad range of antidepressant drugs is one of the most important features supporting its primary use as a screen in antidepressant discovery research. Clinically effective treatments for depression that are detected by the rat FST include: tricyclics, monoamine oxidase inhibitors, atypical antidepressants, therefore, it has been used as a reliable screen test for antidepressant drug development (Cryan et al. 2005b). In this study, we found that immune-regulatory GF showed antidepressant effects, without showing hyperactivity (Figure 5(A,B)). Although the mice consumed more food in the GF-treated groups, but the weight of the mice in the GF-treated groups were not significantly changed (Figure 6(A,C)). Thus, GF extracts might be of use in the clinical treatment of mood disorders.

GF demonstrated stronger antidepressant effects in comparison with PO. Different components in a mushroom extract may have different or even synergistic activities (Vickers 2002; Borchers et al. 2004). There have been several reports of mushrooms containing more than one polysaccharide with antitumor activity. The responses to different polysaccharides are likely to be mediated by different cell surface receptors, which may be present only in specific subsets of cells.

Several polysaccharides purified from GF demonstrate anticancer, immune enhancing efficacy (Kodama et al. 2002, 2004). A chemically sulphated polysaccharide (S-GAP-P) derived from the water-insoluble polysaccharide of GF mycelia inhibited SGC-7901 cell growth in a dose-dependent manner and induced cell apoptosis (Nie et al. 2006). Cui et al. (2007) investigated the biological functioning of a novel polysaccharide-peptide GFPPS1b, isolated from cultured mycelia of *G. frondosa* GF9801. GFPS1b showed antitumor activity which significantly inhibited the proliferation of human gastric adenocarcinoma (SGC-7901 cells) and it slightly influenced the growth of human normal liver (L-02) cell line. D-fraction isolated from GF enhanced, rather than suppressed, the development of collagen-induced arthritis (CIA) (Shigesue et al. 2000). Administration of D-fraction stimulated the immune functioning of normal and tumour-bearing mice (Kodama et al. 2004).

*Pleurotus ostreatus* (PO) is also a mushroom with medical potential. Lavi et al. (2006) reported that an aqueous polysaccharide extract from PO induces anti-proliferative and pro-apoptotic effects in HT-29 colon cancer cells. A novel water-soluble polysaccharide (POPS-1), which was obtained from the fruiting bodies of PO showed progressively higher anti-tumour activity against HeLa tumour cells *in vitro*, in a dose-dependent manner (Tong et al. 2009).

Whether certain metabolites enhance or suppress immune responses depends on a number of factors, including the nature of the polysaccharide, route of administration, and timing of administrations of the compound in question. The type of activity these metabolites exhibit can also depend on their mechanism of action or the site of activity. Taken together, the present data suggest that GF demonstrates stronger antidepressant effects in comparison to PO (Figure 7(A,B)). This may be due to the differences in the potency of the effective components regulating key biofunctions in the animals. Identifying the effective component(s) which show the antidepressant effects should be one of our future directions.

AMPA receptor signalling regulated by GF might be essential for in its robust antidepressant effects. Previous studies showed that lithium, proteo-β-glucan and dextromethorphan exerted an antidepressant effects in mice in the TST and FST via the up-regulation of AMPA receptor subunits; and an AMPA inhibitor was able to block these antidepressant effects (Gould et al. 2008; Nguyen & Matsumoto 2015; Bao et al. 2016). Recently, AMPA receptors are identified as the central mediator for the pathophysiology and treatment of depression (Bleakman et al. 2007; Freudenberg et al. 2015). AMPA receptor modulators have been used as possible antidepressants in animal models and clinical trials. Recent clinical trials have shown that Org 26576 (ionotropic AMPA-type glutamate receptor enhancer) significantly improves symptoms in depressed patients as revealed by the Montgomery-Asberg Depression Rating Scale (Nations et al. 2012). The biaryl-propyl-sulfonamide ARPs (LY392098 and LY451616, AMPA receptor potentiators) show antidepressant effects in animal models of depression, in learned-helplessness models of depression and in animals exposed to the chronic mild stress procedure (Li et al. 2001; Alt et al. 2005). We found that AMPA antagonist GYKI 52466 was able to block the GF-induced antidepressant effects (Figure 8), suggesting that GF used a common pathway, which is enhancement of AMPA synaptic transmission for this antidepressant effects. This discovery has a big impact on the development of novel antidepressant by using agents or even food-enhancing AMPA receptor signalling for the treatment of depression.

GF is a safe and edible mushroom, but may have fewer side effects than the currently used antidepressants. Potentially, patients may just eat GF as a food supplement for the treatment of depression. This discovery also helps to develop effective and safe drugs for the symptoms of major depressive disorder.

## References

[CIT0001] Adachi Y, Okazaki M, Ohno N, Yadomae T. 1994 Enhancement of cytokine production by macrophages stimulated with (1->3)-beta-D-glucan, grifolan (GRN), isolated from *Grifola frondosa*. Biol Pharm Bull. 17:1554–1560. 7537572 10.1248/bpb.17.1554

[CIT0002] Alt A, Witkin JM, Bleakman D. 2005 AMPA receptor potentiators as novel antidepressants. Curr Pharm Des. 11:1511–1527. 15892659 10.2174/1381612053764814

[CIT0003] Autry AE, Adachi M, Nosyreva E, Na ES, Los MF, Cheng PF, Kavalali ET, Monteggia LM. 2011 NMDA receptor blockade at rest triggers rapid behavioural antidepressant responses. Nature. 475:91–95. 21677641 10.1038/nature10130 PMC3172695

[CIT0004] Bai SK, Lee SJ, Na HJ, Ha KS, Han JA, Lee H, Kwon YG, Chung CK, Kim YM. 2005 β-Carotene inhibits inflammatory gene expression in lipopolysaccharide-stimulated macrophages by suppressing redox-based NF-kappaB activation. Exp Mol Med. 37:323–334. 16155409 10.1038/emm.2005.42

[CIT0005] Bao H, Ran P, Zhu M, Sun L, Li B, Hou Y, Nie J, Shan L, Li H, Zheng S, et al 2016 The prefrontal dectin-1/AMPA receptor signaling pathway mediates the robust and prolonged antidepressant effect of proteo-β-glucan from Maitake. Sci Rep 6:28395 27329257 10.1038/srep28395 PMC4916609

[CIT0006] Beneyto M, Kristiansen LV, Oni-Orisan A, McCullumsmith RE, Meador-Woodruff JH. 2007 Abnormal glutamate receptor expression in the medial temporal lobe in schizophrenia and mood disorders. Neuropsychopharmacology. 32:1888–1902. 17299517 10.1038/sj.npp.1301312

[CIT0007] Bhattacharya A, Derecki NC, Lovenberg TW, Drevets WC. 2016 Role of neuro-immunological factors in the pathophysiology of mood disorders. Psychopharmacology (Berl). 233:1623–1636. 26803500 10.1007/s00213-016-4214-0

[CIT0008] Bleakman D, Alt A, Witkin JM. 2007 AMPA receptors in the therapeutic management of depression. CNS Neurol Disord Drug Targets. 6:117–126. 17430149 10.2174/187152707780363258

[CIT0009] Borchers AT, Keen CL, Gershwin ME. 2004 Mushrooms, tumors, and immunity: an update. Exp Biol Med (Maywood). 229:393–406. 15096651 10.1177/153537020422900507

[CIT0010] Borsini F, Meli A. 1988 Is the forced swimming test a suitable model for revealing antidepressant activity? Psychopharmacology. 94:147–160. 3127840 10.1007/BF00176837

[CIT0011] Cryan JF, Mombereau C, Vassout A. 2005a The tail suspension test as a model for assessing antidepressant activity: review of pharmacological and genetic studies in mice. Neurosci Biobehav Rev. 29:571–625. 15890404 10.1016/j.neubiorev.2005.03.009

[CIT0012] Cryan JF, Valentino RJ, Lucki I. 2005b Assessing substrates underlying the behavioral effects of antidepressants using the modified rat forced swimming test. Neurosci Biobehav Rev. 29:547–569. 15893822 10.1016/j.neubiorev.2005.03.008

[CIT0013] Cui J, Chisti Y. 2003 Polysaccharopeptides of *Coriolus versicolo*r: physiological activity, uses, and production. Biotechnol Adv. 21:109–122. 14499133 10.1016/s0734-9750(03)00002-8

[CIT0014] Cui FJ, Li Y, Xu YY, Liu ZQ, Huang DM, Zhang ZC, Tao WY. 2007 Induction of apoptosis in SGC-7901 cells by polysaccharide-peptide GFPS1b from the cultured mycelia of *Grifola frondosa* GF9801. Toxicol Vitro. 21:417–427. 10.1016/j.tiv.2006.10.004 17150327

[CIT0015] Deng G, Lin H, Seidman A, Fornier M, D'Andrea G, Wesa K, Yeung S, Cunningham-Rundles S, Vickers AJ, Cassileth B. 2009 A phase I/II trial of a polysaccharide extract from *Grifola frondosa* (Maitake mushroom) in breast cancer patients: immunological effects. J Cancer Res Clin Oncol. 135:1215–1221. 19253021 10.1007/s00432-009-0562-z PMC3751581

[CIT0016] Douglas RJ, Martin KA. 2007 Mapping the matrix: the ways of neocortex. Neuron. 56:226–238. 17964242 10.1016/j.neuron.2007.10.017

[CIT0017] Duric V, Banasr M, Stockmeier CA, Simen AA, Newton SS, Overholser JC, Jurjus GJ, Dieter L, Duman RS. 2013 Altered expression of synapse and glutamate related genes in post-mortem hippocampus of depressed subjects. Int J Neuropsychopharmacol. 16:69–82. 22339950 10.1017/S1461145712000016 PMC3414647

[CIT0018] Farley S, Apazoglou K, Witkin JM, Giros B, Tzavara ET. 2010 Antidepressant-like effects of an AMPA receptor potentiator under a chronic mild stress paradigm. Int J Neuropsychopharmacol. 13:1207–1218. 20059803 10.1017/S1461145709991076

[CIT0019] Freudenberg F, Celikel T, Reif A. 2015 The role of α-amino-3-hydroxy-5-methyl-4-isoxazolepropionic acid (AMPA) receptors in depression: central mediators of pathophysiology and antidepressant activity? Neurosci Biobehav Rev. 52:193–206. 25783220 10.1016/j.neubiorev.2015.03.005

[CIT0020] Ghasemi M, Phillips C, Trillo L, De Miguel Z, Das D, Salehi A. 2014 The role of NMDA receptors in the pathophysiology and treatment of mood disorders. Neurosci Biobehav Rev. 47:336–358. 25218759 10.1016/j.neubiorev.2014.08.017

[CIT0021] Gould TD, O'Donnell KC, Dow ER, Du J, Chen G, Manji HK. 2008 Involvement of AMPA receptors in the antidepressant-like effects of lithium in the mouse tail suspension test and forced swim test. Neuropharmacology. 54:577–587. 18096191 10.1016/j.neuropharm.2007.11.002 PMC2275050

[CIT0022] Ishibashi K, Miura NN, Adachi Y, Ohno N, Yadomae T. 2001 Relationship between solubility of grifolan, a fungal 1,3-beta-d-glucan, and production of tumor necrosis factor by macrophages *in vitro*. Biosci Biotechnol Biochem. 65:1993–2000. 11676011 10.1271/bbb.65.1993

[CIT0023] Kim JH, Na HJ, Kim CK, Kim JY, Ha KS, Lee H, Chung HT, Kwon HJ, Kwon YG, Kim YM. 2008 The non-provitamin A carotenoid, lutein, inhibits NF-kappaB-dependent gene expression through redox-based regulation of the phosphatidylinositol 3-kinase/PTEN/Akt and NF-kappaB-inducing kinase pathways: role of H_2_O_2_ in NF-kappaB activation. Free Radic Biol Med. 45:885–896. 18620044 10.1016/j.freeradbiomed.2008.06.019

[CIT0024] Kodama N, Komuta K, Sakai N, Nanba H. 2002 Effects of D-fraction, a polysaccharide from *Grifola frondosa* on tumor growth involve activation of NK cells. Biol Pharm Bull. 25:1647–1650. 12499658 10.1248/bpb.25.1647

[CIT0025] Kodama N, Murata Y, Nanba H. 2004 Administration of a polysaccharide from *Grifola frondosa* stimulates immune function of normal mice. J Med Food. 7:141–145. 15298759 10.1089/1096620041224012

[CIT0026] Koike H, Iijima M, Chaki S. 2011 Involvement of AMPA receptor in both the rapid and sustained antidepressant-like effects of ketamine in animal models of depression. Behav Brain Res. 224:107–111. 21669235 10.1016/j.bbr.2011.05.035

[CIT0027] Kong E, Sucic S, Monje FJ, Savalli G, Diao W, Khan D, Ronovsky M, Cabatic M, Koban F, Freissmuth M, et al 2015 STAT3 controls IL6-dependent regulation of serotonin transporter function and depression-like behavior. Sci Rep. 5:9009. 25760924 10.1038/srep09009 PMC5390910

[CIT0028] Krystal JH, Sanacora G, Blumberg H, Anand A, Charney DS, Marek G, Epperson CN, Goddard A, Mason GF. 2002 Glutamate and GABA systems as targets for novel antidepressant and mood-stabilizing treatments. Mol Psychiatry. 7:S71–S80. 11986998 10.1038/sj.mp.4001021

[CIT0029] Lavi I, Friesem D, Geresh S, Hadar Y, Schwartz B. 2006 An aqueous polysaccharide extract from the edible mushroom *Pleurotus ostreatus* induces anti-proliferative and pro-apoptotic effects on HT-29 colon cancer cells. Cancer Lett. 244:61–70. 16413114 10.1016/j.canlet.2005.12.007

[CIT0030] Li X, Tizzano JP, Griffey K, Clay M, Lindstrom T, Skolnick P. 2001 Antidepressant-like actions of an AMPA receptor potentiator (LY392098). Neuropharmacology. 40:1028–1033. 11406194 10.1016/s0028-3908(00)00194-5

[CIT0031] Liu YN, Peng YL, Liu L, Wu TY, Zhang Y, Lian YJ, Yang YY, Kelley KW, Jiang CL, Wang YX. 2015 TNFalpha mediates stress-induced depression by upregulating indoleamine 2,3-dioxygenase in a mouse model of unpredictable chronic mild stress. Eur Cytokine Netw. 26:15–25. 26083579 10.1684/ecn.2015.0362 PMC4764086

[CIT0032] Maeng S, Zarate CA Jr., Du J, Schloesser RJ, McCammon J, Chen G, Manji HK. 2008 Cellular mechanisms underlying the antidepressant effects of ketamine: role of alpha-amino-3-hydroxy-5-methylisoxazole-4-propionic acid receptors. Biol Psychiatry. 63:349–352. 17643398 10.1016/j.biopsych.2007.05.028

[CIT0033] Mattila P, Salo-Vaananen P, Konko K, Aro H, Jalava T. 2002 Basic composition and amino acid contents of mushrooms cultivated in Finland. J Agric Food Chem. 50:6419–6422. 12381127 10.1021/jf020608m

[CIT0034] Mengoni ES, Vichera G, Rigano LA, Rodriguez-Puebla ML, Galliano SR, Cafferata EE, Pivetta OH, Moreno S, Vojnov AA. 2011 Suppression of COX-2, IL-1β and TNF-α expression and leukocyte infiltration in inflamed skin by bioactive compounds from Rosmarinus officinalis L. Fitoterapia. 82:414–421. 21129455 10.1016/j.fitote.2010.11.023

[CIT0035] Musazzi L, Treccani G, Mallei A, Popoli M. 2013 The action of antidepressants on the glutamate system: regulation of glutamate release and glutamate receptors. Biol Psychiatry. 73:1180–1188. 23273725 10.1016/j.biopsych.2012.11.009

[CIT0036] Nations KR, Dogterom P, Bursi R, Schipper J, Greenwald S, Zraket D, Gertsik L, Johnstone J, Lee A, Pande Y, et al 2012 Examination of Org 26576, an AMPA receptor positive allosteric modulator, in patients diagnosed with major depressive disorder: an exploratory, randomized, double-blind, placebo-controlled trial. J Psychopharmacol. 26:1525–1539. 22954616 10.1177/0269881112458728

[CIT0037] Nguyen L, Matsumoto RR. 2015 Involvement of AMPA receptors in the antidepressant-like effects of dextromethorphan in mice. Behav Brain Res. 295:26–34. 25804358 10.1016/j.bbr.2015.03.024

[CIT0038] Nie X, Shi B, Ding Y, Tao W. 2006 Preparation of a chemically sulfated polysaccharide derived from *Grifola frondosa* and its potential biological activities. Int J Biol Macromol. 39:228–233. 16822541 10.1016/j.ijbiomac.2006.03.030

[CIT0039] Okazaki M, Adachi Y, Ohno N, Yadomae T. 1995 Structure-activity relationship of (1->3)-beta-D-glucans in the induction of cytokine production from macrophages, in vitro. Biol Pharm Bull. 18:1320–1327. 8593430 10.1248/bpb.18.1320

[CIT0040] Pilc A, Wieronska JM, Skolnick P. 2013 Glutamate-based antidepressants: preclinical psychopharmacology. Biol Psychiatry. 73:1125–1132. 23453290 10.1016/j.biopsych.2013.01.021

[CIT0041] Porsolt RD. 2000 Animal models of depression: utility for transgenic research. Rev Neurosci. 11:53–58. 10716655 10.1515/revneuro.2000.11.1.53

[CIT0042] Porsolt RD, Le Pichon M, Jalfre M. 1977 Depression: a new animal model sensitive to antidepressant treatments. Nature. 266:730–732. 559941 10.1038/266730a0

[CIT0043] Sanacora G, Treccani G, Popoli M. 2012 Towards a glutamate hypothesis of depression: an emerging frontier of neuropsychopharmacology for mood disorders. Neuropharmacology. 62:63–77. 21827775 10.1016/j.neuropharm.2011.07.036 PMC3205453

[CIT0044] Sanzen I, Imanishi N, Takamatsu N, Konosu S, Mantani N, Terasawa K, Tazawa K, Odaira Y, Watanabe M, Takeyama M, et al 2001 Nitric oxide-mediated antitumor activity induced by the extract from *Grifola frondosa* (Maitake mushroom) in a macrophage cell line, RAW264.7. J Exp Clin Cancer Res. 20:591–597. 11876556

[CIT0045] Shen KP, Su CH, Lu TM, Lai MN, Ng LT. 2015 Effects of *Grifola frondosa* non-polar bioactive components on high-fat diet fed and streptozotocin-induced hyperglycemic mice. Pharm Biol. 53:705–709. 25431253 10.3109/13880209.2014.939290

[CIT0046] Shigesue K, Kodama N, Nanba H. 2000 Effects of maitake (*Grifola frondosa*) polysaccharide on collagen-induced arthritis in mice. Jpn J Pharmacol. 84:293–300. 11138730 10.1254/jjp.84.293

[CIT0047] Steru L, Chermat R, Thierry B, Simon P. 1985 The tail suspension test: a new method for screening antidepressants in mice. Psychopharmacology (Berl). 85:367–370. 3923523 10.1007/BF00428203

[CIT0048] Tong H, Xia F, Feng K, Sun G, Gao X, Sun L, Jiang R, Tian D, Sun X. 2009 Structural characterization and *in vitro* antitumor activity of a novel polysaccharide isolated from the fruiting bodies of *Pleurotus ostreatus*. Bioresour Technol. 100:1682–1686. 18954976 10.1016/j.biortech.2008.09.004

[CIT0049] Vickers A. 2002 Botanical medicines for the treatment of cancer: rationale, overview of current data, and methodological considerations for phase I and II trials. Cancer Invest. 20:1069–1079. 12449740 10.1081/cnv-120005926

